# mRNA Profiles of Porcine Parathyroid Glands Following Variable Phosphorus Supplies throughout Fetal and Postnatal Life

**DOI:** 10.3390/biomedicines9050454

**Published:** 2021-04-22

**Authors:** Michael Oster, Henry Reyer, Christian Gerlinger, Nares Trakooljul, Puntita Siengdee, Jonas Keiler, Siriluck Ponsuksili, Petra Wolf, Klaus Wimmers

**Affiliations:** 1 Institute of Genome Biology, Leibniz Institute for Farm Animal Biology (FBN), 18196 Dummerstorf, Germany; oster@fbn-dummerstorf.de (M.O.); reyer@fbn-dummerstorf.de (H.R.); gerlinger@fbn-dummerstorf.de (C.G.); trakooljul@fbn-dummerstorf.de (N.T.); siengdee@fbn-dummerstorf.de (P.S.); ponsuksili@fbn-dummerstorf.de (S.P.); 2 Department of Anatomy, Rostock University Medical Center, 18057 Rostock, Germany; Jonas.Keiler@med.uni-rostock.de; 3 Faculty of Agricultural and Environmental Sciences, University Rostock, 18059 Rostock, Germany; petra.wolf@uni-rostock.de

**Keywords:** parathyroid function, early nutrition, mineral metabolism, monocalcium phosphate, phosphorus intake

## Abstract

Knowledge of gene expression profiles reflecting functional features and specific responsiveness of parathyroid glands (PTGs) contributes to understanding mineral homeostasis and parathyroid function in healthy and diseased conditions. The study aims to reveal effector molecules driving the maintenance of phosphorus (P) homeostasis and parathyroid hormone (PTH) responsiveness to variable P supply throughout fetal and postnatal life. In this study, a long-term dietary intervention was performed by keeping pig offspring on distinct mineral P levels throughout fetal and postnatal life. Respective adaptation processes of P homeostasis were assessed in mRNA profiles of PTGs and serum minerals. RNA sequencing data and resulting molecular pathways of PTGs showed that the PTH abundance is very strictly controlled via e.g., *PIN1*, *CaSR*, *MAfB*, PLC and PKA signaling to regulate PTH expression, stability, and secretion. Additionally, the observed dietary effects on collagen expression indicate shifts in the ratio between connective tissue and parenchyma, thereby affecting cell-cell contacts as another line of PTH regulation. Taken together, the mRNA profiles of porcine PTGs reflect physiological responses in-vivo following variable dietary P supplies during fetal and postnatal life. The results serve to evaluate a long-term nutrition strategy with implications for improving the mineral balance in individuals with pathological disorders.

## 1. Introduction

The maintenance of the mineral homeostasis is essential for ensuring the physical integrity of all vertebrates. Therefore, endogenous regulatory processes balance the absorption, utilization and excretion of phosphorus (P) and calcium (Ca), whose availability and storage are important for a number of biological processes, in first line bone development [1]. Endogenous adaptive mechanisms targeting the maintenance of mineral homeostasis could compensate a low dietary P supply to certain extents [2,3,4]. However, early life periods are considered vulnerable to dietary challenges [5], which has been summarized in the concept of ‘Nutritional Programming’ [6,7]. It basically claims that a structure or tissue become permanently altered due to physiological responses to external stimuli such as a dietary pattern in early life [8]. Nutritional Programming might offer possible explanations for the onset of metabolic disorders [9], but also provide opportunities to promote endogenous mechanisms towards an efficient nutrient utilization and resource allocation. Regarding mineral efficiency, effective alterations might affect the gastrointestinal tract, bones and kidneys but also the parathyroid glands (PTGs).

The PTGs are essential in restoring hypocalcaemia by secretion of parathyroid hormone (PTH). The superior endocrine control allows mobilizing Ca from body reserves such as hydroxyapatite in bone via stimulated osteoclastic activity [10]. Since Ca and P are in stoichiometric equilibrium, bone resorption triggered by PTH also mobilizes P. Consequently, PTH prevents systemic P excess in serum through increasing renal P excretion via the parathyroid hormone 1 receptor (PTH1R) signaling [11]. Additionally, PTH acts on renal 1α-hydroxylase (CYP27B1), the key enzyme for the hydroxylation of calcidiol as the storage form of vitamin D. The active form of vitamin D formed in the kidney, calcitriol, in turn increases intestinal mineral absorption [12].

To ensure availability of PTH in mammals, PTGs are composed primarily of chief cells and an age-dependent number of oxyphil cells [13]. Both cell types have been shown to be capable to release PTH and to respond to Ca levels [14]. With regard to expression, stability and secretion of PTH, detailed mechanisms have already been described that revealed the sophisticated interaction of PTGs with dynamic physiological conditions and circulating serum levels of e.g., vitamin D, fibroblast growth factor 23 (FGF23), Ca, and P. First, calcitriol acts on PTGs via a Vitamin D Response Element (VDRE) motif in the promoter region of PTH [15], thereby decreasing PTH mRNA levels, and promoting intestinal mineral absorption. Second, PTGs perceive levels of FGF23, a phosphaturic hormone released by osteoblasts and osteocytes, via the parathyroid receptors FGFR1 and α-klotho [16]. Third, PTGs transduce information about current circulating Ca levels in serum via the calcium sensing receptor (CaSR) and downstream signaling cascades to modify PTH release [17,18]. Interestingly, novel binding sites for both Ca and possibly P have been identified at the extracellular domain of the human CaSR [18]. Indeed, extracellular P sensing in vertebrates has proven to be difficult to assess [19]. Nevertheless, serum P levels were associated with the transmission of post-transcriptional regulation to stabilize PTH mRNA copies [19,20]. Moreover, transcription factors such as Gata3, GCM2 and MafB as well as a number of miRNAs are discussed to orchestrate intracellular signaling pathways that modify transcription, mRNA stability, protein synthesis, and release of PTH [19,21,22,23]. Overall, it is known that PTGs respond to dietary-induced hypophosphatemia by altering the expression, mRNA stability, and release of PTH, although detailed mechanisms are not yet fully understood.

A feeding experiment according to the concept of ‘Nutritional Programming’ was conducted, in which sows and their offspring were supplied with three different levels of dietary mineral P throughout pregnancy, lactation and postnatal life. Thus, offspring have experienced variable P supply during both fetal and postnatal life, where amounts of pre- and postnatal dietary P (i) were maintained, (ii) reduced, or (iii) increased considering current recommendations [24]. Respective adaptation processes of P homeostasis were assessed in mRNA profiles of PTGs and serum minerals. The comprehensive molecular characterization of PTGs in pigs aims to reveal effector molecules driving the maintenance of P homeostasis and PTH responsiveness to variable P supply throughout fetal and postnatal life.

## 2. Materials and Methods

### 2.1. Diets, Animals and Sample Collection

The trial was approved by the Animal Welfare Committee of the FBN and was licensed by the Ethics Committee of the federal state of Mecklenburg-Western Pomerania, Germany (LALLF 7221.3-1-053/15; date of approval: 16 December 2015). The experimental protocol comprised 14 nulliparous German Landrace sows (Figure S1) which were randomly assigned to three dietary groups as described previously [25]. The individually fed sows were housed in two batches (*n* = 6; *n* = 8), each representing animals of all three feeding groups. Eight of the 14 sows were unrelated. The remaining six sows were originated from two litters (3 full sibs each). Full sibs have been evenly distributed among the experimental feeding groups.

Sows were artificially inseminated with semen from two German Landrace boars (one boar per sow batch). For an adaptation period (10 days) and throughout the whole gestation (115 days) and lactation (28 days), sows were fed iso-energetic standard corn-barley based diets with recommended amounts of P (M_ges_, M_lac_; *n* = 4), or a diet with reduced (L_ges_, L_lac_; *n* = 5) or higher P content (H_ges_, H_lac_; *n* = 5). Except for P, formulated diets met requirements [24] and did not contain additional phytase supplements of microbial origin (Table S1). Daily feed intake was restricted to 2.8 kg (early gestation), 3.2 kg (late gestation), 2.0 kg (term), and 6.5 kg (lactation) fed in two rations per day. Water was supplied ad libitum. Pregnant sows were transferred to individual farrowing pens at day 109, where the sows remained with their litter during the lactation period. No cross-fostering was applied. The offspring of both German Landrace boars was distributed across all dietary groups.

From day 14 of life, piglets were offered starter diets (st) containing variable amounts of P in accordance to the respective maternal dietary P supply (M_st_, L_st_, H_st_; Table S2). At weaning (day 28), each three male and three female offspring per litter were randomly assigned to one of three standard grower (gr) diets from day 29 to 62 and standard fattener (fat) diets from day 63 to 120 with recommended (M_gr_, M_fat_), reduced (L_gr_, L_fat_) or higher amounts of P (H_gr_, H_fat_). This resulted in nine experimental groups of offspring, each with one of three different maternal (e.g., L_mat_ receiving L_ges_ and L_lac_) and one of three different post-weaning (pw) dietary exposures (e.g., L_pw_, receiving L_gr_ and L_fat_). The feeding trial comprised 84 offspring (Figure S1). One pig each of the groups L_mat_M_pw_ and M_mat_L_pw_ died postnatally. Water was supplied ad libitum. Pigs were kept in groups of 8–10 individuals in pens equipped with concrete floor. In total, samples of 40 males and 42 females have been used. Blood samples of pigs were taken from the jugular vein at day 119 (*n* = 82). Serum was prepared and stored at −80 °C until further analyses. At day 120, pigs were stunned by electronarcosis and subsequently exsanguinated in the experimental facility of the FBN. The slaughtering comprised five batches à 15–17 pigs. The PTGs were sampled as described previously [26]. The head was detached from each of the carcasses and the PTGs were dissected from the cranial thymus near the carotid bifurcation. During preparation, the thymus tissue surrounding the PTGs was completely removed. No apparent differences in volume or size of PTGs were noted. In total, a complete set of both left and right PTGs were retrieved from 63 individuals and stored at −80 °C until further processing (L_mat_L_pw_, *n* = 7; L_mat_M_pw_, *n* = 6; L_mat_H_pw_, *n* = 8; M_mat_L_pw_, *n* = 5; M_mat_M_pw_, *n* = 7; M_mat_H_pw_, *n* = 7; H_mat_L_pw_, *n* = 7; H_mat_M_pw_, *n* = 8; H_mat_H_pw_, *n* = 8). For mRNA-Seq, samples of 29 males and 34 females have been used. Moreover, samples of M. longissimus dorsi (MLD) originated at the 13th and 14th rib level were retrieved.

### 2.2. Histology

Histology sections from PTGs were generated as previously described [26]. The PTGs were washed in phosphate-buffered saline and fixed overnight with 3.7% buffered paraformaldehyde. Following a dehydration step, PTGs were embedded in paraffin. Staining of 5 µm thin sections was performed with hematoxyline and eosin (HE) and Azan trichrome stain. Microphotographs were generated with a bright field microscope (Leica DM6, Wetzlar, Germany).

### 2.3. Growth and Performance

For a period of 7 weeks, individual values for body weight and feed intake were recorded weekly. Values for daily feed intake (DFI), average daily gain (ADG), and feed conversion ratio (FCR) were calculated. The ash content of the longissimus dorsi muscle (MLD) was determined in triplicate by calcination in a muffle furnace at 600 °C using established protocols [27].

### 2.4. Serum Parameters of P Homeostasis

Serum samples were used to quantify selected parameters of P homeostasis. Levels of inorganic P, Ca, magnesium (Mg) and the activity of alkaline phosphatase (ALP) were analyzed with commercial assays using Fuji DriChem (FujiFilm, Minato, Japan).

### 2.5. RNA Isolation and RNA Sequencing

Tissue material was ground into powder in liquid nitrogen. Whole left and right PTGs of each animal were used for RNA isolation by TRI reagent according to the manufacturer′s directions (Sigma-Aldrich, Taufkirchen, Germany). Subsequently, Baseline-ZERO DNase treatment was applied to RNA extracts (Biozym, Hessisch Oldendorf, Germany). Samples were purified using the column-based NucleoSpin RNA II-Kit (Macherey-Nagel, Düren, Germany). The quantification of RNA was performed with a NanoDrop ND-2000 (Peqlab, Erlangen, Germany). The RNA integrity numbers (RIN) were assessed by a 2100 Bioanalyzer instrument (Agilent Technologies, Santa Clara, CA, USA) and ranged from 6.5 to 8.8 for all samples. The RNA was stored at −80 °C until further use. The successful dissection of PTGs was confirmed by the quantification of PTH expression. In brief, single-stranded cDNA was synthesized and transcript levels of PTH and RPL32 (ribosomal protein L32) as a reference gene (Table S3) were quantified by qRT-PCR as described previously [26]. The samples showed a PTH expression of 6.4 ± 3.1 million copies (mean ± SD). Samples were used for downstream analyses and RNA library preparation according to the TruSeq Stranded mRNA protocol (Illumina, San Diego, CA, USA). An Agilent DNA-1000 chip kit was used for library quality assessment on the Bioanalyzer 2100 platform. Paired-end reads of 101 bp in length were retrieved by RNA sequencing on an Illumina HiSeq2500 instrument (Illumina, San Diego, CA, USA). Data are available from the EMBL-EBI (www.ebi.ac.uk/arrayexpress (accessed on 18 April 2021)) database via the accession number E-MTAB-9768.

### 2.6. Data Analyses

Estimated marginal means (EMM) of phenotypic data and serum measurements were retrieved in R language (v4.0.0; R Core Team, Vienna, Austria) by the package emmeans (v1.4.8). Data were subjected to a linear model (package lmerTest, v3.1-2) and effects of sex, family (sow) as well as dietary group were considered. Differences were considered significant at *p* ≤ 0.05.

In the present study, 63 libraries of PTGs retrieved from pigs following variable P supplies in a full sibling design were analyzed by high-throughput mRNA sequencing. The raw mRNA sequencing data were quality checked and pre-processed. Low quality reads (a mean Q-score < 20) and adapters were removed via FastQC (v0.11.7) and Trim Galore (v0.5.0). The retrieved high quality reads were subsequently mapped to the reference genome Sscrofa11.1 (Ensembl release 93). Gene features were processed via HISAT2 (v2.1.0) [28] and HTSeq (v0.12.4) [29]. One sample of the MH group was identified as outlier by the R-package arrayQualityMetrics (v3.44.0) and excluded from the analysis [30]. A filtering step to remove very low abundant transcripts was applied using DESeq2 (v3.4.0) [31] in R language. Regularized log (rlog) transformed data were used for Spearman correlation analysis. Relative changes of mRNA abundances were estimated via a linear model including dietary group, sex and sow. False discovery rates (FDR) were calculated to correct for multiple testing. The level of significance was set at FDR ≤ 0.05. The lists of differentially expressed genes (DEGs) were used to generate PCA plots based on maternal diet and post-weaning diet, respectively (mixOmics v6.12.2). The expression of key genes involved in mineral homeostasis was used to generate heat maps and summarized as deviation from the mean expression value (rlog) for each dietary group (RColorBrewer v1.1.2, gplots v3.0.3). Moreover, the DEG lists were used for enrichment analysis via Ingenuity Pathway Analyses (IPA). Pathways attributed with the IPA terms ‘cancer’ and ‘disease’ were discarded. To predict activation or inhibition state of the identified pathways, an absolute z-score ≥ 2 provided by IPA was taken into account. Pathways were considered significant with a Benjamini-Hochberg adjusted *p* ≤ 0.05.

## 3. Results

The design of the feeding trial comprised three dietary P supplies for porcine progeny during (i) fetal and early postnatal development (maternal diets; mat), and (ii) post-weaning (pw) until fattening (Figure S1). Therefore, the study deals with nine experimental groups, each with one of three different maternal as well as one of three different post-weaning dietary exposures. The current study assesses long-term transcriptional effects on parathyroid glands (PTGs), associated serum minerals and growth data. Serum has been collected at day 119 of life and PTGs have been sampled at day 120 of life.

### 3.1. Growth and Performance Data

Throughout the fattening period, the values for final body weight (BW), daily feed intake (DFI), average daily gain (ADG), and feed conversion ratio (FCR) were significantly affected by the dietary P supply received after weaning (Table 1, Figure S2). In particular those animals that received the L_pw_ diet showed reduced (final BW, DFI, ADG) or increased (FCR) values. However, the ash content in muscle samples remained unchanged due to dietary P supply. Variable P content of the maternal diets revealed no effect on performance data. However, offspring of low P fed mothers tend to be superior to offspring of other sow groups in all traits when receiving a low P diet themselves, implying subtle conditioning to cope with their own limited P supply (Table 1 and Table 2).

### 3.2. Parameters of P Homeostasis in Serum

At day 119, serum concentrations of inorganic P (IP), Ca, and alkaline phosphatase activity (ALP) were measured to reflect dietary effects on mineral homeostasis. Serum parameters were significantly affected by the dietary P supply received post-weaning (Table 2, Figure S2). The variable P content of the maternal diets revealed no effect on serum parameters. Magnesium (Mg) levels remained unaltered due to dietary P supply.

### 3.3. Verification of PTG Sampling

**Figure 1:**
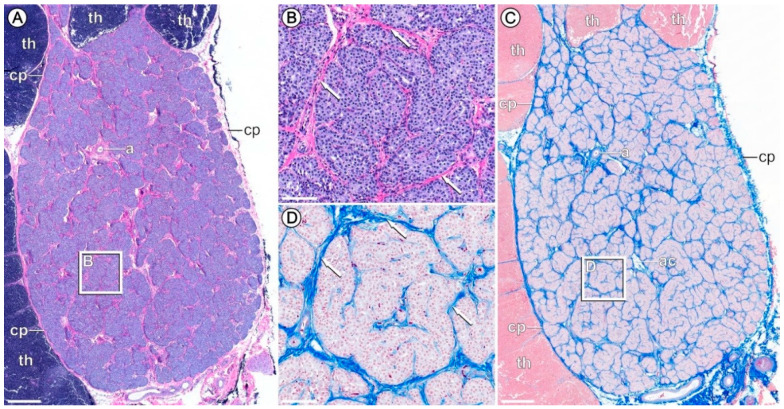
Representative histological sections of a porcine parathyroid gland (PTG). (**A**,**B**) HE staining. (**C**,**D**) Azan trichrome staining. White rectangles refer to magnifications shown in (**B**,**D**). Arrows in magnified areas in (**B**,**D**) depict PTG parenchyma formed by chief cells. Clusters of chief cells are separated by collagenous septa (arrows). Scale bars: 500 µm (**A** + **C**), 100 µm (**B** + **D**). a—artery; ac—adipocytes; cp—capsule; th—thymus.

The dissection of PTGs was verified via histochemical staining of representative samples (Figure 1). Accordingly, PTGs exhibited the characteristic capsule of collagenous connective tissue and a lobule structure. The representative histological sections of porcine PTGs showed the absence of thymus structures in the 120-day-old pigs. The parenchyma consists largely of chief cells. Furthermore, the results on *PTH* gene expression among RNA seq (rlog normalized) and qPCR data showed a highly significant correlation (rho = 0.81; *p* < 0.001).

### 3.4. Transcriptome Profiling of PTGs

Gene expression profiles of PTGs, as a central component of the regulation of mineral status, were analyzed in a holistic manner to reflect the impacts of the P-divergent diets. After filtering, the analyses of the porcine PTGs comprised a set of 12,752 transcripts. The genes showing the highest expression in porcine PTGs are parathyroid secretory protein 1 (*CHGA*; chromogranin A) and PTH with 1.57 million and 1.52 million copies (base mean expression). The genes *A2M*, *ATP6*, *CTSB*, *CYTB*, *COX1*, *COX2*, *COX3*, *EEF1A1*, and FAXDC2 were also among the highest expressed genes with a base mean expression between 107,000 and 714,000 (Tables S4 and S5).

Variation of the maternal P supply, to which the offspring were exposed during the entire gestation and lactation period, led to 36 responsive genes (Table S4). The DEGs could not be assigned to any enriched pathways. According to the consulted annotation tools, the maternal dietary P supply affected transcripts associated with immunity (*JCHAIN*, *OAS2*, *TNFRSF17*), cell integrity (*MXRA5*, *PPP2R2B*, *SHC1*), and the intermediary metabolism (*FCN2*, *FOLH1B*, *GCNT4*, *GYG2*). Results suggest that the effect of the maternal P level on gene expression in PTGs of their offspring was rather secondary (Figure S3).

The divergent dietary P supply post-weaning resulted in a significantly altered expression of 1671 genes (Table S5). It appeared that the post-weaning low-P diet was the main driver on gene expression in PTGs (Figure S3). Enrichment analyses via IPA identified 17 significantly enriched pathways (Table 3). The results pointed to an involvement of DEGs in signal transduction from CaSR (‘Phospholipase C signaling’) and G proteins (‘G-Protein coupled receptor signaling’, ‘Gαi signaling’, ‘cAMP-mediated signaling’, ‘Protein kinase A signaling’, ‘RhoGDI signaling’, ‘α-Adrenergic Signaling’). These results address therefore a number of downstream signaling cascades for PTH expression, stability and secretion. Moreover, highlighted pathways comprised collagen expression and platelet aggregation (‘GP6 signaling pathway’, ‘Thrombin signaling’), immune aspects (‘LPS/IL-1 mediated inhibition of RXR function’) and neuronal development (‘Axonal guidance signaling’). Indeed, 15 of the 17 differentially expressed collagens found in PTGs were elevated in L_pw_-fed animals compared with the medium and high P fed individuals (Table S5). Further significantly enriched pathways are ‘Cardiac hypertrophy signaling’, ‘Cardiac β-adrenergic signaling’, ‘Ephrin B signaling’, ‘Adipogenesis pathway’, ‘Glycogen degradation III’, and ‘NRF2-mediated oxidative stress response’. The predicted activation state (z-score) of the identified pathways revealed a significant activation of ‘GP6 signaling pathway’ (z-score = 2.99), ‘Cardiac hypertrophy signaling’ (z-score = 3.58), ‘Cardiac β-adrenergic signaling’ (z-score = 2.67), and ‘cAMP-mediated signaling’ (z-score = 2.12). The z-scores suggest that functional adaptation due to post-weaning diets is likely with respect to e.g., collagen expression and PTH regulation via cAMP.

**Figure 2:**
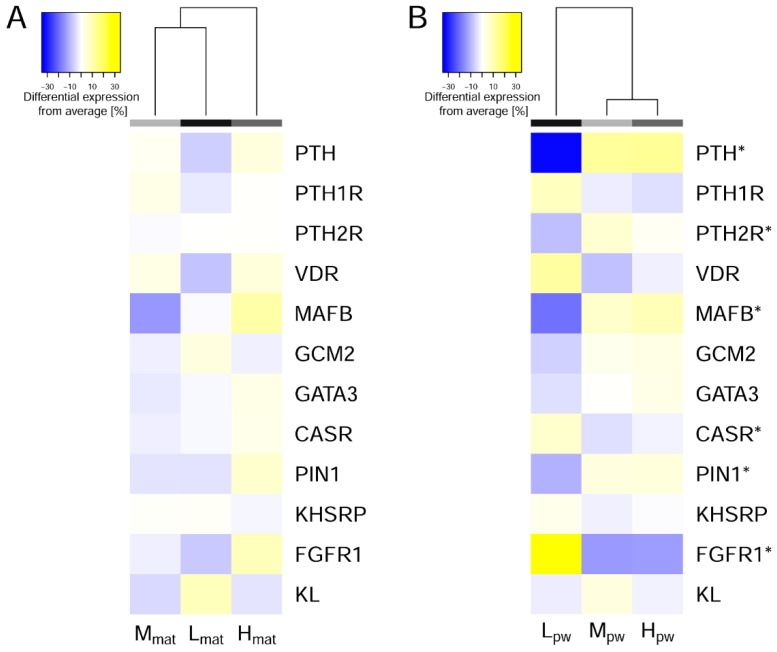
Heat maps of selected key genes expressed in porcine PTGs with implication in systemic mineral homeostasis. Data were presented for dietary effects mediated by (**A**) maternal and (**B**) post-weaning periods. * Genes have been identified as significantly differentially expressed due to variable dietary P supply (Table S5). mat—maternal; pw—post-weaning.

Relative diet-specific expression values of key genes involved in the maintenance of systemic mineral homeostasis were presented relative to average expression levels in PTGs (Figure 2) and as individual dot plots (Figure S4). None of the selected genes showed effects due to the intra-uterine and early postnatal exposure to maternal diets. In contrast, the variable P supply post-weaning showed various endogenous responses, which led to lower expression of *MAfB*, *PIN1*, *PTH*, *PTH2R* and increased expression of *CaSR* and *FGFR1* in the animals fed L_pw_ diets compared to the respective mean expression levels of all investigated animals (Table S5).

## 4. Discussion

Knowledge of the molecular composition and specific responsiveness of PTGs contributes to understanding mineral homeostasis and parathyroid function in health and disease. This study provides for the first time holistic mRNA profiles of porcine PTGs that reflect the physiological conditions in-vivo following variable dietary P supplies during fetal and postnatal life.

### 4.1. Gene Expression Patterns at Physiological Conditions of Porcine PTGs

In mammals, the main function of the PTGs are transcription, storage and release of PTH in order to maintain the serum Ca and P concentrations, despite varying physiological conditions or external stimuli such as dietary mineral supply. Indeed, porcine PTGs appeared to express 12,752 genes, where *PTH* was second in terms of base mean expression. Interestingly, the gene exhibiting the highest copy number in PTGs, the glycoprotein *CHGA*, is known to be co-expressed with PTH and represents an effector molecule within the neuroendocrine system and immunity where it constitutes a series of bioactive peptides [32]. However, its detailed function in PTGs is still unknown. The high base mean expression of *CHGA* and *PTH* serves as a marker for parathyroid cells and thus demonstrates the validity of sampling. Moreover, *A2M* (alpha-2-macroglobulin) showed a high mRNA abundance in PTGs. It is believed that its main function involves inactivating a number of proteases. On the other hand, *CTSB* (cathepsin B) was one of the top expressed genes in PTGs, which is known to play an essential role in the proteolysis of components of the extracellular matrix. The high abundance of both *A2M* and *CTSB* might contribute to balance protein integrity and cleavage, which might comprise another line of PTH regulation according to metabolic demands. In addition, mitochondrial genes involved in oxidative phosphorylation (e.g., *COX1*, *COX2*, *COX3*) were found among the most prominently abundant transcripts, focusing on ensuring energy equivalents for e.g., hormone production. Notably, *CYP27B1*, which encodes the 1α-hydroxylase to catalyze the hydroxylation of calcidiol, was expressed in porcine PTGs at low levels, which could account for non-renal calcitriol production (Table S5).

### 4.2. Endogenous Responses to Balance PTH Expression, Stability and Secretion

The post-weaning effects of the low P supply resulted in a clear reduction in base mean expression of *PTH* mRNA compared to animals fed M_pw_ and H_pw_ diets (FDR < 0.001; Table S5, Figure 2). This corresponds to reduced PTH serum levels that have been observed in pigs fed low P diets [33,34]. Both annotated PTH receptors, *PTH1R* (FDR = 0.067) and *PTH2R* (FDR = 0.003), are moderately expressed in porcine PTGs (Table S5, Figure S4). The *PTH1R* is known to be expressed in a number of tissues such as kidney and bone to transduce PTH effects by molecular signaling cascades. *PTH2R* seems to be expressed with low mRNA abundance in a number of tissues and appears to be highly specific in ligand recognition. The reduced mRNA abundance of *PTH2R* in the PTGs of animals fed the low P diet post-weaning suggest a role of PTH2R in a feedback loop for reporting systemic PTH levels. There is an increasing body of evidence that expression, stability and secretion of PTH are comprehensively sensed and targeted via a vast number of molecules including (i) calcitriol, (ii) transcription factors such as MAFB, and (iii) post-transcriptional modifications including CaSR signaling [23,35].

First, pigs fed a low P diet compared to a diet according to the current recommendations (0.28% soluble P vs. 0.43% soluble P) showed significantly elevated serum calcitriol levels (537 ± 19 pmol/l vs. 335 ± 74 pmol/l; mean ± SEM) at 63 days of life [2]. While vitamin D receptor (*VDR*) expression in PTGs was unaffected by diets (FDR = 0.066), elevated calcitriol levels in L_pw_ pigs reflect a response to maintain physiological serum P levels due to known calcitriol-mediated effects on *PTH* mRNA abundance via negative VDRE [15].

Second, by exploiting the expression of selected key genes with a pronounced effect on mineral homeostasis (Figure 2 and Figure S4), the reduced mRNA abundance of the transcription factor *MAFB* (FDR = 0.005) could further contribute to the lowered *PTH* mRNA levels, although the expression of its co-regulators *GCM2* (FDR = 0.459) and *GATA3* (FDR = 0.577) remained unaffected by dietary P supply [36].

Third, serum P levels were found reduced in the offspring fed the low P diet post-weaning compared to the other groups (Table 2). Notably, serum P concentrations were identified as a post-transcriptional signal that modulate the *PTH* mRNA stability [37,38]. This involves CaSR as a key factor in mineral sensing [18], whose mRNA abundance in PTGs responded to the post-weaning diets (FDR = 0.029) and showed slightly elevated values in animals kept on the low P diet post-weaning. The *CaSR* is expressed in chief cells [39] and oxyphil cells [14] but also plays an essential role in C-cells of the thyroid and in the kidney tubules, where its expression is known to be sensitive to diet in pigs and rats [40,41].

Moreover, the gene expression profile of porcine PTGs revealed *Pin1* (FDR = 0.024) to be affected by post-weaning diets, which showed a lowered mRNA abundance in animals fed the L_pw_ diet. As Pin1 is a key molecule to destabilize the *PTH* mRNA by a 3′UTR cis-acting element in P-depleted conditions [42], the *Pin1* expression in this study suggests a line of compensatory regulation. However, the expression of its co-regulator *KHSRP* was unaffected by post-weaning diets (FDR = 0.418).

The orchestration to maintain the P homeostasis mentioned above is complemented by FGF23 which favors a decreased *PTH* expression [16]. The serum FGF23 status is sensed by the parathyroid glands via fibroblast growth factor receptor 1 (FGFR1) and its co-receptor α-klotho (KL). While the mRNA copy number of *KL* was unaffected (FDR = 0.401), the *FGFR1* mRNA abundance was responsive to post-weaning diets and significantly increased in animals which have received low P diets (Table S5). Notably, cyclins such as *CCND1* and *CCND2* were unaffected by diets which accounts for a non-proliferative state of the PTGs. In contrast, findings derived from experimental models and patients with secondary hyperparathyroidism (SHP) following chronic kidney disease (CKD) showed a decrease in parathyroid cell proliferation as reviewed elsewhere [38]. Results of PTG expression profiles demonstrate the resilience to dietary P reductions via endogenous compensatory responses towards Ca/P homeostasis via strictly balancing *PTH* regulation at the level of mRNA expression and stability, synthesis and tissue sensitivity.

### 4.3. Gene Expression Patterns at Physiological Conditions of Porcine PTGs

The expression profiles of porcine PTGs showed a number of pathways, which primarily show physiological responses to the L_pw_ diet (Table 3). In fact, the identified pathways illustrate the transduction of systemic signals to the intracellular level and represent the adaptive coordination to maintain long-term Ca/P homeostasis in the offspring. The signaling events associated with G protein-coupled receptors such as CaSR are therefore prominent. Consequently, the signaling pathway of phospholipase C (PLC) is addressed in porcine PTGs (Table 3) as a downstream effector for an inhibitory action on PTH regulation [43]. At the same time, animals fed the L_pw_ diet showed an activated cAMP-mediated signaling cascade, which prerequisites the phosphokinase A (PKA) signaling pathway (Table 3) towards positive PTH regulation. The results suggest that the PLC and PKA pathways in porcine PTGs are carefully balanced, which is consistent with the precise regulation of PTH expression, stability and release via previously reported metabolites, receptors and positive and negative enhancers. This requires complex communication between the different cell types present in the PTGs. The anatomic architecture of PTGs is reflected by a high expression of collagens as an important modulatory structure affecting the degree of compartmentalization and subsequently the cell-cell contacts between chief cells [19,44]. Accordingly, animals fed the low P diet post-weaning showed an activated GP6 signaling pathway (Table 3) with a number of collagens exhibiting increased mRNA abundances (Table S5). It remains to be clarified whether this could also be responsible for a higher proportion of collagens at the protein level and alterations in the organization of cell-cell contacts between the chief cells in L_pw_ animals. Given the reduced *PTH* mRNA expression in L_pw_ animals, the increased mRNA abundances of collagens support the concept of paracrine interaction for PTH regulation [45]. The putative role of collagen abundance to control PTH production resembles findings on the involvement of myofibroblasts in human parathyroid glands as reported previously [46]. In the current pig study, the expression of the myofibroblast markers such as vimentin (*VIM*; FDR = 0.003) and alpha smooth muscle actin (*ACTA2*; FDR = 0.002) have been significantly increased in animals which have received the low P diet. The analysis shows evidence of an increased ratio between connective tissue and parenchyma, which may ultimately reduce cell-cell contacts after a low-P diet post-weaning. Accordingly, there is a need to illustrate dietary responses via immunohistochemical staining and a detailed 3D analysis of the tissue structure including the chief cell densities and the collagen fiber configuration.

### 4.4. Early Life Conditioning and Implications on Long-Term Health

The serum mineral levels and gene expression profiles analyzed in PTGs represent post-weaning dietary responses rather than maternal effects (Figure S3). After 120 days of postnatal life the effect of maternal variable P supply on the offspring appeared superimposed by the dietary post-weaning challenge. The results show the hierarchically superior role of PTGs in regulating serum Ca concentrations with emphasis on compensating for acute nutritional mineral imbalances. However, the maternal low P diet during lactation revealed increased endogenous calcitriol production at day 28 compared to maternal M_mat_ and H_mat_ diets [25]. Offspring of sows fed on the low P diet are likely to have received higher levels of calcitriol via milk during lactation [47], which could have negatively influenced *PTH* mRNA abundances in porcine PTGs. Consequently, at day 120 variations in serum P and Ca levels matched physiological ranges [48]. Actually, the offspring of L-fed sows seem to be rather able to adjust their serum P level when they themselves received a low P supply compared to a medium or higher P supply post-weaning (Table 2). It remains to be clarified whether a more pronounced variation in maternal P supply would be effective on the transcriptional or epigenetic level.

## 5. Conclusions

Taken together, dietary long-term effects on expression profiles of porcine PTGs provide information on physiological responses to low P intake and indicate synergistic regulation of feedback loops for systemic control of PTH expression and stability. The tested maternal P intakes appeared to have minor effects on offspring PTG transcriptome patterns, whereas the variable dietary P supply post-weaning effectively induced physiological endogenous responses to maintain P homeostasis. However, the blood P and Ca levels were not completely balanced, but remained within physiological ranges. The data revealed that porcine PTGs are responsive to external stimuli, which highlights the pig to serve as a model for human conditions. The molecular characterization of the porcine PTGs at physiological conditions will allow the identification of new strategies to reduce the diagnostic burden in individuals with mineral imbalances.

## Figures and Tables

**Table 1:** Growth and performance data of animals exposed to maternal (mat) and post-weaning (pw) diets with recommended (M), reduced (L) or higher (H) mineral P contents. Data were retrieved from individual pigs for a period of 7 weeks until day 119.

Maternal Diet	Offspring Diet	BW [kg]	DFI [kg/d]	ADG [kg/d]	FCR [g/g]	Ash (MLD) [%]
L	L	60.72	1.67	0.74	2.27	1.20
L	M	69.30	1.88	0.85	2.18	1.19
L	H	65.54	1.73	0.81	2.14	1.20
M	L	47.70	1.30	0.60	2.16	1.22
M	M	58.09	1.56	0.74	2.08	1.22
M	H	62.19	1.67	0.81	2.07	1.20
H	L	55.30	1.50	0.68	2.18	1.20
H	M	58.17	1.54	0.73	2.11	1.22
H	H	62.83	1.62	0.80	2.02	1.20
Pooled SEM		9.00	0.28	0.11	0.22	0.03
*p*-Value	Maternal diet	0.327	0.453	0.117	0.798	0.657
	post-weaning diet	<0.001	0.015	<0.001	0.015	0.640
	Mat diet × pw diet	0.207	0.188	0.193	0.941	0.302

BW—body weight; DFI—daily feed intake; ADG—average daily gain; FCR—feed conversion ratio; MLD—M. longissimus dorsi.

**Table 2:** Serum data referring to P homeostasis for animals exposed to maternal (mat) and post-weaning (pw) diets with recommended (M), reduced (L) or higher (H) mineral P contents. Data were retrieved from individual pigs at day 119.

Maternal Diet	Offspring Diet	IP [mg/dL]	Ca [mg/dL]	Mg [mg/dL]	ALP [U/L]
L	L	8.94	10.97	2.21	164.8
L	M	10.75	10.21	2.20	135.1
L	H	10.66	10.40	2.12	158.4
M	L	8.05	11.44	2.22	237.5
M	M	10.66	10.54	2.14	142.8
M	H	10.60	10.42	2.11	153.3
H	L	8.66	11.04	2.22	169.0
H	M	11.28	10.64	2.24	144.9
H	H	10.75	10.52	2.20	143.6
Pooled SEM		0.90	0.57	0.20	36.5
*p*-Value	Maternal diet	0.305	0.517	0.902	0.053
	Post-weaning diet	<0.001	<0.001	0.327	<0.001
	Mat diet × pw diet	0.346	0.466	0.873	0.011

IP—inorganic phosphate; Ca—calcium; Mg—magnesium; ALP—alkaline phosphatase activity.

**Table 3:** Canonical pathways based on DEGs identified from PTG expression profiles of porcine offspring exposed to maternal and post-weaning diets with recommended (M_pw_), reduced (L_pw_) or higher (H_pw_) mineral P contents.

Canonical Pathway	Adjusted *p*-Value	z-Score ^†^ (L_pw_ vs. H_pw_)	z-Score ^†^ (L_pw_ vs. M_pw_)	z-Score ^†^ (M_pw_ vs. H_pw_)
Axonal guidance signaling	0.0003	-	-	-
Protein kinase A signaling	0.0015	1.07	1.07	−1.07
RhoGDI signaling	0.0016	1.15	1.15	−0.69
GP6 signaling pathway	0.0016	2.99	2.99	0.43
Cardiac hypertrophy signaling (enhanced)	0.0016	3.58	3.58	0.66
NRF2-mediated oxidative stress response	0.0021	−1.04	−1.04	−1.04
Phospholipase C signaling	0.0021	0.78	0.78	0.39
Cardiac β-adrenergic signaling	0.0021	2.67	2.67	−0.73
cAMP-mediated signaling	0.0021	2.12	2.12	0.00
LPS/IL-1 mediated inhibition of RXR function	0.0032	0.71	0.71	0.71
Thrombin signaling	0.0042	1.21	1.21	0.24
Adipogenesis pathway	0.0046	-	-	-
Glycogen degradation III	0.0068	−0.82	−0.82	0.82
α-Adrenergic signaling	0.0083	0.91	0.91	−1.51
G-Protein coupled receptor signaling	0.0083	-	-	-
Ephrin B signaling	0.0093	−1.00	−1.00	0.33
Gαi signaling	0.0093	1.50	1.50	0.50

^†^ z-score: pathways with an absolute z-score ≥ 2 were considered significant. Positive and negative values indicate activation (e.g., L_pw_ > M_pw_) and inhibition (e.g., L_pw_ < M_pw_). pw—post-weaning. -: dashes indicate that no calculation could be made.

## Data Availability

All raw data of deep sequencing have been deposited in the EMBL-EBI (www.ebi.ac.uk/arrayexpress (accessed on 18 April 2021)) database as E-MTAB-9768.

## Supplementary Materials

The following are available online at https://www.mdpi.com/article/10.3390/biomedicines9050454/s1, Figure S1: Experimental design. Three diets with recommended (M), reduced (L), or higher (H) mineral P contents were fed to pregnant sows (*n* = 14) for an initial adaptation period and throughout gestation (115 days) and lactation (28 days). Balanced for sex, six piglets per litter were assigned to three diets differing in P contents at weaning (day 28) until slaughter (120 days of life). Offspring were exposed to long-term variations in dietary P supplies throughout gestation (ges), lactation (lac), growing (gr) and fattening (fat) periods which generated nine experimental groups. No cross-fostering was applied. Samples of offspring were retrieved at 119 days (serum, *n* = 82) and 120 days (parathyroid glands, *n* = 63). I—insemination; F—farrowing; W—weaning; S—sampling. Figure S2: Individual performance trait data (live weight, DFI, ADG, FCR, ash content in MLD) and serum traits (P, Ca) for animals exposed to maternal (mat) and post-weaning (pw) diets with recommended (M), reduced (L), or higher (H) mineral P content are presented as a combined dot plot and boxplot. The shape of dots refer to the respective maternal diet. Colors refer to the respective post-weaning diet. Figure S3. PCA plots of gene expression data (rlog) of parathyroid glands derived from animals exposed to maternal (A) and post-weaning diets (B). Individual animals are represented by colors which refer to recommended (M), reduced (L), or higher (H) mineral P contents, respectively. DEG—differentially expressed gene. Figure S4. Individual gene expression data for animals exposed to maternal (mat) and post-weaning (pw) diets with recommended (M), reduced (L), or higher (H) mineral P content. Expression data is based on rlog transformation and is presented as a combined dot plot and boxplot. Black solid lines represent mean values calculated for animals exposed to the same maternal dietary P levels. The shape of dots refer to the respective maternal diet. Colors refer to the respective post-weaning diet. Table S1: Analyzed nutrient composition of the experimental diets fed to sows during gestation and lactation, Table S2: Analyzed nutrient composition of the experimental diets fed to offspring during starter, grower and fattener periods, Table S3: Primer used in qRT-PCR to verify dissection of PTGs, Table S4: Maternal effects on gene expression in porcine parathyroid glands (PTGs). List of transcripts derived from porcine offspring exposed to maternal and postnatal diets with recommended (Mmat), reduced (Lmat) or higher (Hmat) mineral P contents (csv). Associated statistics refer to maternal dietary effects (base mean expression, P value, FDR, fold change). Table S5: Post-weaning effects on gene expression in porcine parathyroid glands (PTGs). List of transcripts derived from porcine offspring exposed to maternal and postnatal diets with recommended (M_pw_), reduced (L_pw_) or higher (H_pw_) mineral P contents (csv). Associated statistics refer to maternal dietary effects (base mean expression, *p* value, FDR, fold change).
