# Supplementary material for: mRNA Profiles of Porcine Parathyroid Glands Following Variable Phosphorus Supplies throughout Fetal and Postnatal Life
Source: Biomedicines. 2021 Apr 22;9(5):454. doi: 10.3390/biomedicines9050454 (PMC8146947; doi:10.3390/biomedicines9050454)
Supplement: Supplementary file 1 [file biomedicines-09-00454-s001.zip › Table S1_Diet-composition_sows.pdf]

Table S1. Analyzed nutrient composition of the experimental diets fed to sows during gestation and lactation.

		**Gestation diets**			**Lactation diets**		
**Item**	**Unit**	**L_{ges}**	**M_{ges}**	**H_{ges}**	**L_{lac}**	**M_{lac}**	**H_{lac}**
DM	g/kg	889	888	890	888	898	887
Crude ash	g/kg	45.1	49.4	48.2	51.4	60.7	67.0
Crude protein	g/kg	142	154	145	189	195	198
Crude fat	g/kg	27.1	30.3	23.7	29.6	25.8	24.0
Crude fibre	g/kg	87.8	89.4	86.7	61.9	56.8	52.2
Starch	g/kg	282	268	284	388	385	391
ME (pig)	MJ/kg	11.2	11.2	11.1	12.5	12.7	12.6
Calcium	g/kg	6.55	6.95	6.70	7.20	7.30	7.10
Phosphorus (soluble)	g/kg	3.70	4.60	5.55	4.80	6.10	7.20
Ca:P ratio		1.77	1.51	1.21	1.50	1.20	0.99

ges – gestation diet; lac – lactation diet
