# Supplementary material for: mRNA Profiles of Porcine Parathyroid Glands Following Variable Phosphorus Supplies throughout Fetal and Postnatal Life
Source: Biomedicines. 2021 Apr 22;9(5):454. doi: 10.3390/biomedicines9050454 (PMC8146947; doi:10.3390/biomedicines9050454)
Supplement: Supplementary file 1 [file biomedicines-09-00454-s001.zip › Table S2_Diet-composition_offspring.pdf]

Table S2. Analyzed nutrient composition of the experimental diets fed to offspring during starter, grower and fattener periods.

**Diets**		**Starter diets (day 14-28)**			**Grower diets (day 29-62)**			**Fattener diets (day 63-120)**		
**Item**	**Unit**	L_{st}	M_{st}	H_{st}	L_{gr}	M_{gr}	H_{gr}	L_{fat}	M_{fat}	H_{fat}
DM	g/kg	936	95	935	904	904	887	880	881	883
Crude ash	g/kg	76.9	75.2	81.3	64.3	67.0	62.6	38.9	40.3	44.6
Crude protein	g/kg	218	220	215	188	185	179	178	179	175
Crude fat	g/kg	128	126	125	54.4	55.7	52.6	28.8	30.3	31.2
Crude fibre	g/kg	17.6	26.8	21.4	48.1	42.8	36.1	34.9	40.0	38.7
Starch	g/kg	174	150	156	373	375	378	434	428	432
ME (pig)	MJ/kg	16.0	15.7	15.7	13.5	13.7	13.6	13.5	13.4	13.4
Calcium	g/kg	9.60	8.61	8.99	10.60	10.02	9.59	6.50	6.40	6.70
Phosphorus	g/kg	6.63	7.88	9.11	5.65	6.84	7.95	4.12	5.50	6.96
Ca:P ratio		1.45	1.09	0.99	1.88	1.46	1.21	1.58	1.16	0.96

st – starter diet; gr – grower diet; fat – fattener diet
