# Supplementary material for: mRNA Profiles of Porcine Parathyroid Glands Following Variable Phosphorus Supplies throughout Fetal and Postnatal Life
Source: Biomedicines. 2021 Apr 22;9(5):454. doi: 10.3390/biomedicines9050454 (PMC8146947; doi:10.3390/biomedicines9050454)
Supplement: Supplementary file 1 [file biomedicines-09-00454-s001.zip › Table S3_primer-qPCR.pdf]

Table S3. Primer used in qRT-PCR to verify dissection of PTGs.

Primer	Sequence 5' – 3'	Melting temp. (°C)	Amplicon length (bp)
PTH (for)	CAGCCTTCTCGGGTTTACTCA	59.6	228
PTH (rev)	TCAGGTGTTTGCCCAGGTTA	58.7	
RPL32 (for)	AGCCCAAGATCGTCAAAAAG	57.1	165
RPL32 (rev)	TGTTGCTCCCATAACCAATG	56.6	

PTH – parathyroid hormone; RPL32 – ribosomal protein L32;
