# Supplementary figures and images for: mRNA Profiles of Porcine Parathyroid Glands Following Variable Phosphorus Supplies throughout Fetal and Postnatal Life
Source: Biomedicines. 2021 Apr 22;9(5):454. doi: 10.3390/biomedicines9050454 (PMC8146947; doi:10.3390/biomedicines9050454)

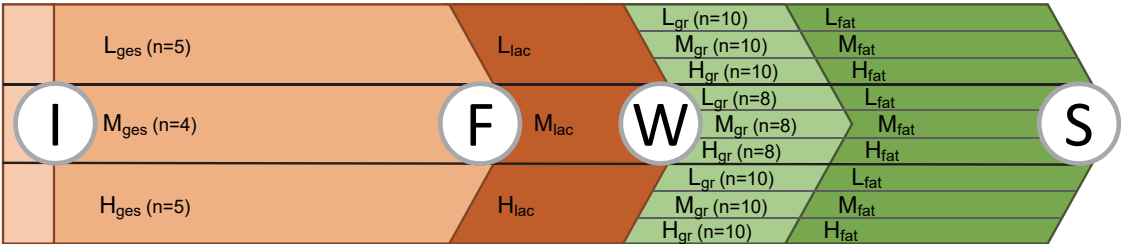

Gestation diet

Lactation diet

Grower diet

Fattener diet

Supplement: Supplementary file 1 [file biomedicines-09-00454-s001.zip › Figure S1_ExDesign.pdf]

A

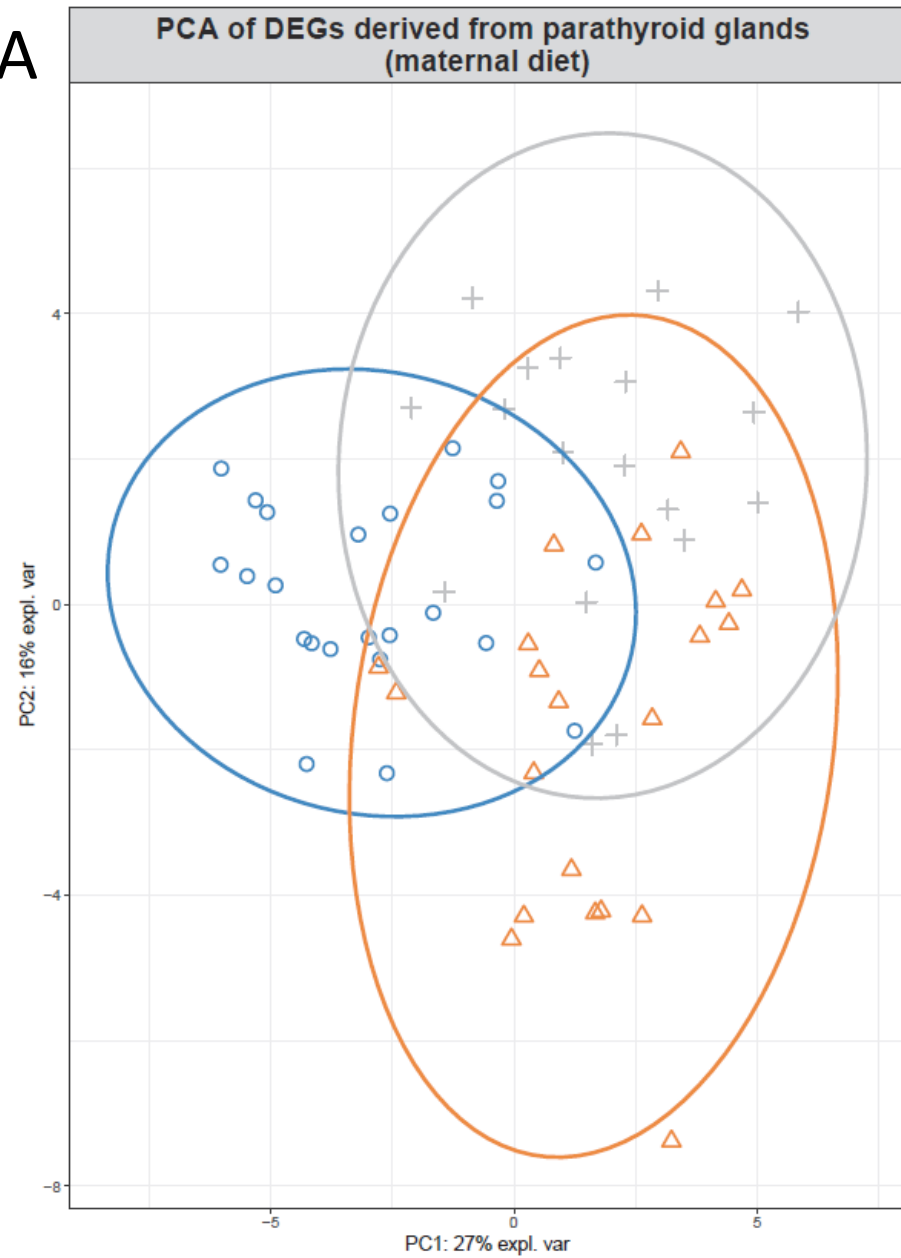

B

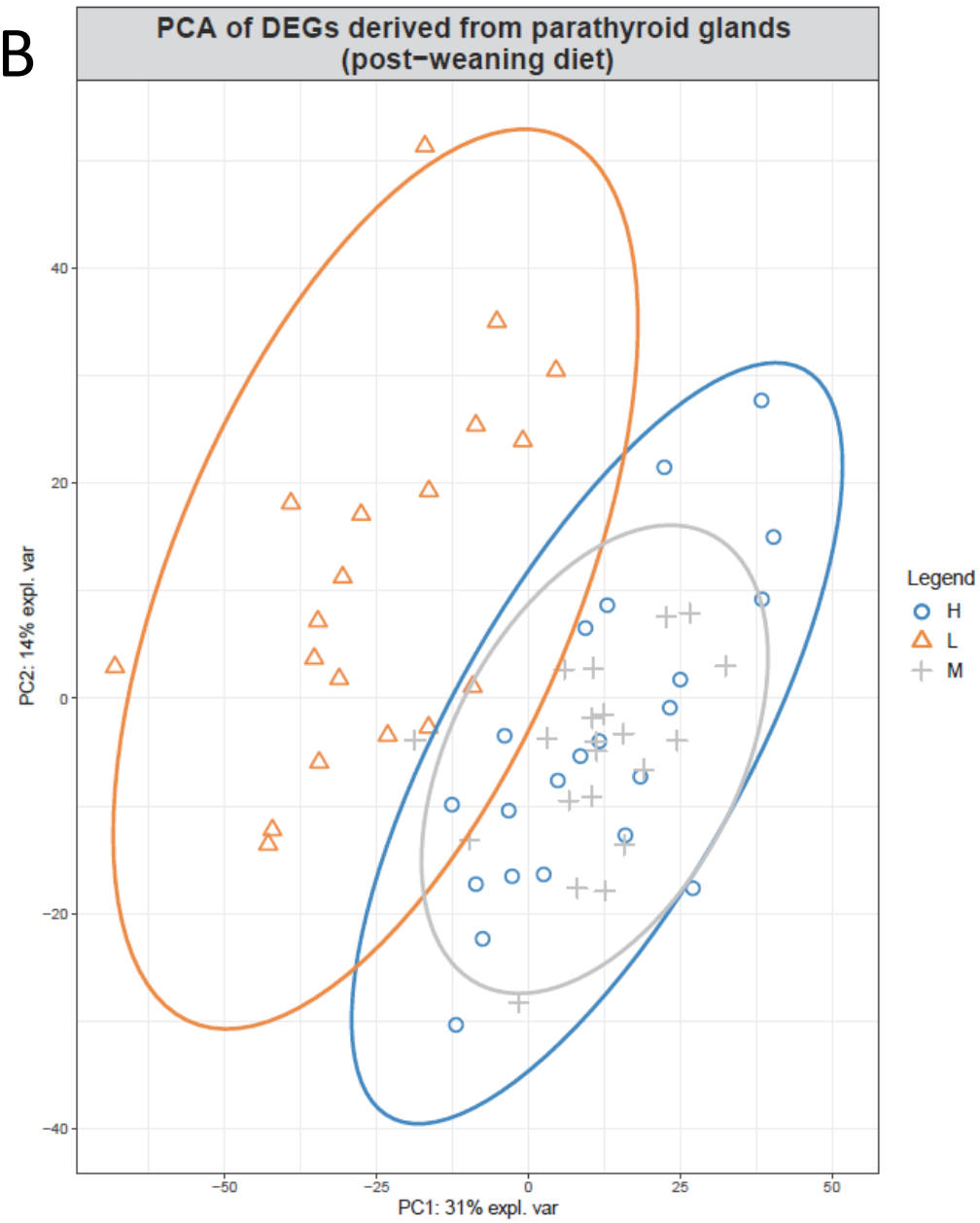

Supplement: Supplementary file 1 [file biomedicines-09-00454-s001.zip › Figure S3_PCA-plots_expression.pdf]
